# Methicillin-Resistant *Staphylococcus aureus* in Marine Mammals

**DOI:** 10.3201/eid1512.090220

**Published:** 2009-12

**Authors:** Meredith C. Faires, Erica Gehring, June Mergl, J. Scott Weese

**Affiliations:** University of Guelph, Guelph, Ontario, Canada (M.C. Faires, J.S. Weese); Niagara Falls Animal Medical Centre, Niagara Falls, Ontario, Canada (E. Gehring, J. Mergl)

**Keywords:** methicillin-resistant Staphylococcus aureus, transmission, humans, dolphins, walruses, staphylococci, letter

**To the Editor:** Methicillin-resistant *Staphylococcus aureus* (MRSA) is emerging as an important cause of illness and death in animals and has been found in an impressive variety of species. However, to date, only 2 studies have reported the isolation of MRSA from marine mammals, 1 seal (*1*) and 3 bottlenose dolphins (*2*). We describe an investigation that was conducted after MRSA was isolated from a dolphin at a marine park in North America.

In November 2006, a 20-year-old, male, captive, bottlenose dolphin, suspected of having pneumonia, was treated empirically with ciprofloxacin and itraconazole. Despite treatment, the dolphin died in December 2006. A necropsy was performed, and a culture swab specimen of the blowhole was submitted for bacteriologic examination; MRSA was then isolated. The clinical relevance of this finding was unclear. Pulsed-field gel electrophoresis (PFGE) was conducted (*3*), and results indicated that the MRSA strain isolated was the Canadian epidemic MRSA (CMRSA)2 (USA100) strain, the predominant hospital- and community-associated MRSA strain found in persons in Canada (*4*). To determine the extent of MRSA colonization in this marine park, blowhole swab specimens were collected from dolphins, orcas, and beluga whales, and nasal swab specimens were collected from walruses, sea lions, harbor seals, gray seals, and park personnel, excluding 4 employees in January 2007. Selective culture for MRSA was performed, and strains were typed with PFGE (*3*) and *spa* typing (*5*). All MRSA strains were investigated for the Panton-Valentine leukocidin (PVL) toxin genes (*6*).

In January 2007, MRSA was not isolated from personnel (0/22), sea lions (0/12), harbor seals (0/2), gray seals (0/2), orcas (0/4), or beluga whales (0/23); it was isolated from dolphins (2/6, 33.3%) and a walrus (1/6, 16.7%). To reduce the risk for MRSA transmission among the marine mammals and to personnel, the following steps were recommended: colonized animals were isolated, contact with colonized animals was restricted, all park personnel were required to wear gloves and masks when handling colonized animals, and routine hand hygiene was emphasized. Colonized walruses were isolated in a separate facility until May 2007. Because of space limitations, colonized dolphins could not be isolated. Although the park instituted a strict policy that required personnel to wear gloves and masks, this policy ceased during the summer months due to the park’s exhibition schedule.

Because we knew from our observations of other animal species that natural decolonization with MRSA is common, as well as lacking information about antimicrobial drug efficacy for MRSA decolonization in marine mammals, and had concerns regarding the emergence of further antimicrobial drug resistance, we recommended that no attempt be made to decolonize the animals with antimicrobial agents. After these recommendations were made and implemented, follow-up testing for MRSA colonization was performed on the dolphins and walruses throughout 2007 and 2008 (Table). In October 2007, testing conducted on all sea lions, harbor seals, gray seals, orcas, and beluga whales showed that none of these animals were colonized with MRSA. Overall, MRSA was isolated on >1 occasions from 5 dolphins (n = 6, 83.3%) and 3 walruses (n = 6, 50%) (Table). All strains were indistinguishable on PFGE and were consistent with the CMRSA2 (USA100) strain. They were also *spa* type t002 and did not possess the PVL toxin genes.

**Table:** MRSA colonization status of dolphins and walruses during 2007–2008*

Date	No. (%) dolphins MRSA positive	Identification nos. of MRSA-positive dolphins	No. (%) walruses MRSA positive	Identification nos. of MRSA-positive walruses
2007 Jan	2/6 (33.3)	2, 3	1/6 (16.7)	1
2007 Feb	2/6 (33.3)	2, 4	2/5 (40)	2, 3
2007 Apr	2/5† (40)	3, 5	0/6 (0)	NA
2007 May	2/3 (66.7)	3, 5	0/6	NA
2007 Oct	1/5 (20)	3	0/5	NA
2008 May	1/5 (20)	3	NT	NA
2008 Jul	0/5	NA	NT	NA
2008 Oct	0/5	NA	NT	NA

*MRSA, methicillin-resistant *Staphylococcus aureus;* NA, not applicable; NT, not tested. †Dolphin 2 died due to unknown circumstances.

This report of MRSA shows colonization in several dolphins and walruses, with apparent transmission between species. The direction of transmission cannot be determined because of the sampling method; however, a human origin is suspected because the clone that was isolated is a predominant human clone. The failure to identify a concurrently colonized person does not preclude a human source. Since the time MRSA was introduced into the facility is unknown, the source of infection may have been decolonized by the time of sampling or was not sampled. Furthermore, park visitors occasionally have contact with these animals so the origin could have been from the general public. Whether colonization of multiple animals was due to repeated instances of human-to-animal transmission or whether animal-to-animal transmission may have occurred is not clear. For the dolphins, the second scenario is most likely, considering the social nature of these animals and the inability to isolate colonized dolphins. These factors may have resulted in the circulation of MRSA among these animals. Although no water samples were obtained for testing, waterborne transmission cannot be dismissed.

Colonization was eliminated without antimicrobial agents; however, long-term (15 months) MRSA colonization was found in 1 dolphin. With patience and continued use of infection control measures, MRSA was apparently eradicated from this facility without the need for active decolonization. This study shows the impressive ability of MRSA to colonize diverse animal species and provides further evidence suggesting that interspecies transmission of human epidemic clones can occur between persons and animals. This study also provides evidence suggesting that MRSA colonization in many animal species can be transient and that application of appropriate infection control and hygiene measures may be critical control tools for the management of MRSA in animals.

## References

[1] O’Mahony R, Abbott Y, Leonard FC, Markey BK, Quinn PJ, Pollock PJ, Methicillin-resistant *Staphylococcus aureus* (MRSA) isolated from animals and veterinary personnel in Ireland. Vet Microbiol. 2005;109:285–96. 10.1016/j.vetmic.2005.06.003 16026939

[2] Schaefer AM, Goldstein JD, Reif JS, Fair PA, Bossart GD. Antibiotic-resistant organisms cultured from Atlantic bottlenose dolphins (*Tursiops truncatus*) inhabiting estuarine waters of Charleston, SC and Indian River Lagoon, FL. EcoHealth. 2009;6:33–41. 10.1007/s10393-009-0221-5 19415386

[3] Mulvey MR, Chui L, Ismail J, Louie L, Murphy C, Chang N, Development of a Canadian standardized protocol for subtyping methicillin-resistant *Staphylococcus aureus* using pulsed-field gel electrophoresis. J Clin Microbiol. 2001;39:3481–5. 10.1128/JCM.39.10.3481-3485.2001 11574559 PMC88375

[4] Mulvey MR, MacDougall L, Cholin B, Horsman G, Fidyk M, Woods S, Community-associated methicillin-resistant *Staphylococcus aureus*, Canada. Emerg Infect Dis. 2005;11:844–50. 15963278 10.3201/eid1106.041146 PMC3367573

[5] Shopsin B, Gomez M, Montgomery SO, Smith DH, Waddington M, Dodge DE, Evaluation of protein A gene polymorphic region DNA sequencing for typing of *Staphylococcus aureus* strains. J Clin Microbiol. 1999;37:3556–63. 10523551 10.1128/jcm.37.11.3556-3563.1999 PMC85690

[6] Rankin S, Roberts S, O’Shea K, Maloney D, Lorenzo M, Benson CE. Panton valentine leukocidin (PVL) toxin positive MRSA strains isolated from companion animals. Vet Microbiol. 2005;108:145–8. 10.1016/j.vetmic.2005.02.013 15917142

